# Work-family life courses and metabolic markers in mid-life: evidence from the British National Child Development Study

**DOI:** 10.1136/jech-2015-206036

**Published:** 2015-12-12

**Authors:** Anne McMunn, Rebecca E Lacey, Meena Kumari, Diana Worts, Peggy McDonough, Amanda Sacker

**Affiliations:** 1 Department of Epidemiology & Public Health, University College London, London, UK; 2 Institute for Social and Economic Research, University of Essex, Colchester, UK; 3 Dalla Lana School of Public Health, University of Toronto, Toronto, Canada

**Keywords:** Life course epidemiology, EMPLOYMENT, MARITAL STATUS, Cohort studies

## Abstract

**Background:**

Previous studies have found generally better health among those who combine employment and family responsibilities; however, most research excludes men, and relies on subjective measures of health and information on work and family activities from only 1 or 2 time points in the life course. This study investigated associations between work-family life course types (LCTs) and markers of metabolic risk in a British birth cohort study.

**Methods:**

Multichannel sequence analysis was used to generate work-family LCTs, combining annual information on work, partnership and parenthood between 16 and 42 years for men and women in the British National Child Development Study (NCDS, followed since their birth in 1958). Associations between work-family LCTs and metabolic risk factors in mid-life (age 44–45) were tested using multivariate linear regression in multiply imputed data.

**Results:**

Life courses characterised by earlier transitions into parenthood were associated with significantly increased metabolic risk, regardless of attachment to paid work or marital stability over the life course. These associations were only partially attenuated by educational qualifications, early life circumstances and adult mediators. The positive association between weak labour markets ties and metabolic risk was weaker than might be expected from previous studies. Associations between work-family LCTs and metabolic risk factors did not differ significantly by gender.

**Conclusions:**

Earlier transitions to parenthood are linked to metabolic risk in mid-life.

## Introduction

A large body of evidence has shown that employment has health benefits,^1–3^ independent of the negative effects of poor health on labour force participation.^4^ However, work and family circumstances are inextricably entwined, especially—though not exclusively—for women. Transitions to parenthood continue to impact participation in paid work, especially for mothers.^5–9^ In addition, marriage has been linked with increased longevity, improved health outcomes (perhaps more so for men than women) and, of course, parenthood.^10–12^ Studies examining health effects of the interdependence between work and family life have found that combining paid work with family responsibilities is usually associated with health advantages^13–20^ (but see^21–23^), although only a handful of these studies have explicitly accounted for the possible effects of health selection into employment, marriage and parenthood.^13–14^ ^16^ ^18^ Existing research on work-family and health has at least three short comings. First, the frequent exclusion of men^13–14^ ^17–23^ presupposes that work and family domains are independent for men. Yet, we know that married fathers tend to have stronger ties to paid work than single men suggesting that work and family are entwined for both genders.^24^

Second, a life course approach is lacking from much previous evidence with few studies including information about work and family activities from more than two time points. Also, despite strong evidence from life course epidemiology attesting to its importance, the timing of family transitions is not usually considered in studies of work-family and health. Early entry into parenthood, for example, has been associated with worse health^24–29^ in adulthood, although only two of these studies have taken account of the potential effect of health prior to parenthood.^27^ ^29^ The increasing availability of longitudinal life course surveys and new statistical techniques to characterise long-term individual-level data now allow for a more complete picture of work and family activities over the life course, including the timing of work and family transitions. A life course approach is also important because work and family biographies are likely to be influenced by early life social and economic experiences^30^—as well as early health and well-being—all of which are also likely to influence adult health independently.^31^

Third, with a few exceptions,^13^ ^20^ ^22^ most previous research has relied on subjective health measures.^14–19^ ^21^ ^23^ The use of objective markers of health is important for determining whether associations extend beyond mental health to physical health and, if so, for identifying possible explanatory mechanisms. Stress is often proposed as a pathway likely to mediate relationships between work-family life and health.^14^ ^19^ Early studies proposed that stress may result from the multiple demands of combining paid work and parenting activities;^21^ however, the subsequent body of evidence suggests that weak ties to employment and marriage, as well as early family transitions, are more often linked with increased stress.^2^ ^10^ ^11^ ^27^ Part of the stress of these experiences may be their impact on adult socioeconomic circumstances.^27^ ^32^ Exposure to stress may be linked with physical health outcomes, either directly through chronic physiological stress responses, and/or indirectly through risky health behaviours.^25^ ^27^ ^33^

We use multichannel sequence analysis, which allows for the holistic characterisation of multiple-domain trajectories, as work-family life course types (LCTs) using data from the British National Child Development Study (NCDS), a cohort of men and women followed since their birth in 1958. We then investigate the impact of work-family LCTs on metabolic risk factors, biomarkers thought to mediate associations between stress and health.^34^ ^35^ We hypothesise that stressful work-family LCTs, such as those characterised by earlier transitions to parenthood and weak ties to paid work and marriage, may be linked with raised metabolic risk. Following a life course approach, we also examine whether early life circumstances that may set people onto more disadvantaged work-family trajectories explain associations with metabolic risk. Finally, we investigate the mediating roles of health behaviours and occupational class in adulthood.

## Methods

### Data

Our data are from the NCDS which recruited 17 415 babies born in 1 week of 1958 (98.2% of all births that week) in Great Britain.^36^ Data on economic, medical, developmental and social aspects of participants’ lives were collected at birth and ages 7, 11, 16, 23, 33, 42, 44/45, 46 and 50. At age 44/45, a subsample of participants (n=9377, 77.9% of the target) completed a biomedical survey measuring biomarkers, including a range of metabolic risk factors. The NCDS data are publicly available and ethical approval for each wave of data collection was received from a UK Multi-Centre Research Ethics Committee.

### Measures

#### Metabolic risk factors

Six markers of metabolic risk were available at age 44/45: waist circumference (WC), systolic and diastolic blood pressure (SBP and DBP), high-density lipoprotein (HDL) cholesterol, triglycerides and glycated haemoglobin (HbA1c). Triglycerides and HDL cholesterol were log-transformed for analysis as they were positively skewed.

#### Work-family LCTs

Annual work, partnership and parenthood statuses were derived for ages 16 through 42. Work status was defined as full-time employment, part-time employment (≤30 h/week), full-time caring for children at home, or other not employed (unemployed, sick, in education or other). Partnership status was defined as married, cohabiting or not living with a partner. Parental status was categorised as no children in the household or youngest child >16 years, youngest child in household <5 years, or youngest child in household 5–16 years. Values for each age were then cross-classified to create 26 annual work-family state variables, each with 36 possible combinations of work, partnership and parenthood. Multichannel sequence analysis was used to reduce this combination of variables to a set of work-family life course ‘types’ by measuring the distance from each individual's work-family sequence to a set of model work-family biographies. Twelve model sequences were specified based on prior knowledge of this cohort, and with a view to capturing adequate variation across both genders while maintaining sufficient power (table 1). Distances were calculated using the Dynamic Hamming variant of optimal matching analysis,^37^ and participants were categorised based on their closest model biography. Further information on the derivation of these work-family LCTs has been published previously.^38^

**Table 1:** Sample distributions for work-family life course types (LCTs) and associated ‘model biography’ sequences in the National Child Development Study 1958 British birth cohort

Work-family type	Men, % (N=3532)*†	Women, % (N=3696)*†	Model biographies (ages 16–42)
‘Work, Later Family’	34.4	8.9	Continuous full-time employment; cohabiting mid-20s, married from late 20s; parent from early 30s
‘Work, Cohabitation, Later Parent’	6.5	5.1	Continuous full-time employment; cohabiting from mid-20s; parent from early 30s
‘Work, Marriage, Non-Parent’	7.8	8.9	Continuous full-time employment; married from early 20s; no children
‘Work, Earlier Family’	31.9	11.7	Continuous full-time employment; married and parent from early 20s
‘Later Family, Work Break’	0.2	14.0	Employed full-time until late 20s, caring for children full-time from early 30s; married from mid-20s; parent from early 30s
‘Work, No Family’	12.8	10.1	Continuous full-time employment; no partner or children
‘Earlier Family, Work Break’	0.1	15.8	Employed full-time until early 20s, caring for children full-time from early 20s, employed part-time from early 30s; married and parent from early 20s
‘Part-time Work, Earlier Family’	0.3	18.0	Employed full-time until early 20s, part-time employed from early 20s; married and parent from early 20s
‘No Paid Work, Earlier Family’	0.1	3.3	Employed part-time until early 20s, caring for children full-time from early 20s; marriage and parent from early 20s
‘Work, Divorced Parent’	4.2	2.5	Continuous full-time employment; married from early 20s, single from late 30s; parent from early 20s
‘Teen parent’	0.8	1.2	Caring for children full-time until mid-20s, employed full-time from mid-20s; married from early 30s; parent from late teens
‘Unstable Work, No Family’	0.9	0.6	Working intermittently; no partner or children

*Percentages are given as data are imputed therefore Ns vary across imputed data sets.

†Sample restricted to those with at least one outcome.

#### Covariates

Two groups of covariates were included: early life factors (adolescent health, fathers’ occupational class and participants’ own educational attainment), and adult mediators (adult household social class, health behaviours and body mass index (BMI)). Adolescent health was measured by physician reports of whether, at age 16, the respondent had any condition likely to affect employment. The Rutter behaviour scales (both mother and teacher reported) assessed emotional health at age 16. Both scales were standardised as z-scores. Father's occupational class at age 16 used the UK Registrar General's Social Class schema, and respondents’ educational attainment was measured as the highest qualification achieved by age 23. While we generally conceptualise educational attainment as an early life predictor of adult life courses, we recognise that the timing of education and family transitions may be bi-directional.

Adult mediators included the highest occupational class of the cohort member or their partner (where relevant) at age 42, using the same coding as for father's occupational class. Other adult mediators were smoking status (never, ex, current), exercise frequency (regularly yes/no), harmful drinking (2+ on the CAGE questionnaire) and self-reported BMI (correlations with measured BMI from preceding and subsequent waves were 0.74 and 0.82, respectively) at age 42.^i^

### Statistical analysis

#### Missing data and analytic sample

Missing information on work, partnership and parenthood was imputed using a method recommended for overcoming problems of collinearity and inaccurate estimation of missing values when imputing sequence data.^39^ Twenty imputed data sets were created at this stage. Subsequently, missing covariate values were multiply imputed by chained equations.^40^ After imputation, our analytic sample comprised 7826 participants with data on work-family life courses and biomarker outcomes. There was some bias in response to the biomedical follow-up survey: work-family LCTs characterised by weak ties to paid work were most likely to drop out while those characterised by later family transitions were least likely.

#### Regression analyses

Associations between work-family groups and metabolic risk factor outcomes were tested using nested multivariate linear regression models. Model 1 estimated the gender-adjusted associations between work-family LCTs and each outcome. Model 2 added early life factors, and model 3 included potential adult mediators. Gender by work-family LCT interaction terms were not statistically significant, so models were run with men and women combined (and controlling for gender). Results for triglycerides and HDL cholesterol are presented as percentage differences, as these outcomes were log-transformed. Sensitivity analyses were conducted for models predicting HbA1c excluding the 122 people who were on antidiabetic medication, and for HDL cholesterol and triglycerides excluding 105 participants on lipid-lowering drugs.

## Results

### Work-family life courses in the NCDS

Table 1 describes the 12 work-family LTCs and their distribution in the NCDS. Almost all men (98%) were in one of the six work-family LTCs characterised by long-term full-time employment, and nearly two-thirds were in a group which combined long-term full-time employment with stable marriage and parenthood. ‘Work, Later Family’ was the modal work-family group for men (34%), followed closely by ‘Work, Earlier Family’ at 32%. Conversely, fewer than half (47%) of women were in one of the work-family LTCs characterised by long-term full-time employment. The ‘Part-time Work, Earlier Family’ group was most common among women (18%), followed by two career break groups: ‘Earlier Family, Work Break’ (16%) and ‘Later Family, Work Break’ (14%). Similar proportions of men and women were in the ‘Work, Cohabitation, Later Parent’ group (7% and 5%, respectively), the ‘Work, Marriage, Non-Parent’ group (8% of men, 9% of women) and the ‘Work, No Family’ group (13% of men, 10% of women). Only 4% of women were in the ‘No Paid Work, Earlier Family’ group, and few men or women were in groups characterised by marital dissolution, teen parenthood or weak ties to work or family.

### Work-family life courses and metabolic risk factors

#### Gender-adjusted associations

Table 2 shows regression results for metabolic risk factors by work-family LCTs. The ‘Work, Later Family’ group was taken as the reference throughout, as strong ties to paid work and later transitions to stable family life were hypothesised to be the most health enhancing, which was largely borne out. Each of the work-family LCTs characterised by earlier entry into parenthood was associated with significantly increased metabolic risk (model 1). In comparison with those who combined long-term employment with later family transitions, those in the ‘Work, Earlier Family’ group had significantly higher levels of each of the metabolic risk factors. Combining earlier parenthood with weaker ties to paid work also raised metabolic risk levels. Those in the ‘No Paid Work, Earlier Family’ group had significantly higher levels of risk on all but the BP measures, while ‘Part-time Work, Earlier Family’ and ‘Earlier Family, Work Break’ groups had significantly lower levels of HDL cholesterol and higher levels of triglycerides. ‘Teen parents’ had riskier levels on all but SBP, although these associations were only significant at the 5% level for WC and triglycerides, probably because there were few individuals in this group. ‘Work, Divorced Parents’ had significantly higher levels on all outcomes except SBP, DBP and WC. Those with ‘Unstable Work, No Family’ also had large coefficients for everything except the BP measures, but these did not reach statistical significance, most likely due to the small size of this group. Those in the ‘Work, No Family’ group had significantly smaller WC, and ‘Work, Cohabitation, Later Parents’ had significantly higher levels of HbA1c in gender-adjusted models.

**Table 2:** Associations between work-family LCTs and metabolic risk factors in the National Child Development Study 1958 British birth cohort

	Model 1: gender-adjusted		Model 2: model 1+early life factors*		Model 3: model 2+adult mediators†	
**Waist circumference (n=7791)**	**Reg coefficient**	**95% CI**	**Reg coefficient**	**95% CI**	**Reg coefficient**	**95% CI**

Work, Later Family	Ref		Ref		Ref	
Work, Cohabitation, Later Parents	−0.59	−1.83 to 0.66	−1.25	−2.56 to 0.07	−1.26	−2.57 to 0.06
Work, Marriage, Non-Parent	−0.19	−1.29 to 0.90	0.06	−1.10 to 1.21	−0.10	−1.25 to 1.05
Work, Earlier Family	1.98	1.17 to 2.79	1.22	0.37 to 2.07	1.12	0.27 to 1.96
Later Family, Work Break	−0.59	−1.86 to 0.67	−0.48	−1.77 to 0.82	−0.50	−1.80 to 0.79
Work, No Family	−1.01	−1.99 to −0.02	−0.70	−1.75 to 0.34	−0.75	−1.79 to 0.29
Earlier Family, Work Break	1.00	−0.21 to 2.21	0.19	−1.07 to 1.45	−0.02	−1.28 to 1.24
Part-time Work, Earlier Family	0.90	−0.26 to 2.07	0.15	−1.06 to 1.36	0.03	−1.18 to 1.23
No Paid Work, Earlier Family	2.44	0.30 to 4.58	0.43	−1.95 to 2.82	−0.66	−3.08, 1.76
Work, Divorced Parent	0.25	−1.32 to 1.82	−0.48	−2.10 to 1.15	−0.60	−2.23 to 1.03
Teen parent	3.54	0.90 to 6.18	5.06	1.46 to 8.66	4.92	1.35 to 8.48
Unstable Work, No Family	3.57	0.47 to 6.67	2.23	−1.51 to 5.97	2.25	−1.57 to 6.07
R^2^ (%)	23.3		24.5		25.9	

**Systolic blood pressure (n=7789)**	**Reg coefficient**	**95% CI**	**Reg coefficient**	**95% CI**	**Reg coefficient**	**95% CI**

Work, Later Family	Ref		Ref		Ref	
Work, Cohabitation, Later Parents	0.18	−1.46 to 1.82	0.02	−1.73 to 1.76	0.20	−1.51 to 1.92
Work, Marriage, Non-Parent	0.71	−0.75 to 2.16	0.62	−0.93 to 2.16	0.62	−0.89 to 2.13
Work, Earlier Family	2.31	1.24 to 3.38	1.97	0.84 to 3.10	1.40	0.29 to 2.51
Later Family, Work Break	−0.62	−2.28 to 1.05	−0.50	−2.23 to 1.23	−0.25	−1.95 to 1.45
Work, No Family	−0.22	−1.51 to 1.08	−0.11	−1.49 to 1.27	0.22	−1.13 to 1.57
Earlier Family, Work Break	1.37	−0.23 to 2.97	0.93	−0.74 to 2.61	0.70	−0.94 to 2.34
Part-time Work, Earlier Family	1.01	−0.53 to 2.55	0.56	−1.05 to 2.17	0.41	−1.17 to 2.00
No Paid Work, Earlier Family	−0.66	−3.44 to 2.24	−2.03	−5.22 to 1.15	−2.13	−5.28 to 1.02
Work, Divorced Parent	1.33	−0.75 to 3.42	1.15	−1.04 to 3.33	1.02	−1.12 to 3.17
Teen parent	1.33	−2.16 to 4.81	0.82	−1.84 to 5.47	0.24	−4.29 to 4.76
Unstable Work, No Family	1.19	−2.82 to 5.20	−0.01	−4.92 to 4.91	−1.99	−6.94 to 2.96
R^2^ (%)	13.5		13.9		18.4	

**Diastolic blood pressure (n=7789)**	**Reg coefficient**	**95% CI**	**Reg coefficient**	**95% CI**	**Reg coefficient**	**95% CI**

Work, Later Family	Ref		Ref		Ref	
Work, Cohabitation, Later Parents	0.08	−1.05 to 1.22	−0.05	−1.25 to 1.16	0.10	−1.08 to 1.29
Work, Marriage, Non-Parent	0.92	−0.08 to 1.92	0.84	−0.22 to 1.91	0.75	−0.29 to 1.80
Work, Earlier Family	1.41	0.68 to 2.15	1.03	0.25 to 1.81	0.65	−0.11 to 1.42
Later Family, Work Break	−0.43	−1.58 to 0.72	−0.53	−1.73 to 0.67	−0.43	−1.61 to 0.74
Work, No Family	0.09	−0.80 to 0.99	0.0001	−0.95 to 0.95	0.18	−0.75 to 1.12
Earlier Family, Work Break	0.52	−0.58 to 1.62	0.18	−0.98 to 1.34	−0.002	−1.14 to 1.13
Part-time Work, Earlier Family	0.69	−0.37 to 1.76	0.28	−0.83 to 1.40	0.17	−0.93 to 1.26
No Paid Work, Earlier Family	−0.69	−2.66 to 1.29	−1.30	−3.52 to 0.91	−1.45	−3.64 to 0.74
Work, Divorced Parent	0.68	−0.76 to 2.13	0.43	−1.09 to 1.95	0.50	−0.99 to 1.99
Teen parent	2.02	−0.35 to 4.38	1.89	−1.30 to 5.08	1.53	−1.60 to 4.67
Unstable Work, No Family	−0.18	−2.92 to 2.57	−0.69	−4.06 to 2.68	−1.57	−4.95 to 1.81
R^2^ (%)	8.7		9.1		13.8	

**HDL cholesterol (n=6597)**	**Per cent difference**	**95% CI**	**Per cent difference**	**95% CI**	**Per cent difference**	**95% CI**

Work, Later Family	Ref		Ref		Ref	
Work, Cohabitation, Later Parents	0.78	−1.85 to 3.47	1.60	−1.20 to 4.48	1.14	−1.49 to 3.85
Work, Marriage, Non-Parent	1.80	−0.54 to 4.19	1.33	−1.13 to 3.86	0.73	−1.58 to 3.08
Work, Earlier Family	−2.70	−4.34 to −1.04	−1.31	−3.05 to 0.46	−0.03	−1.69 to 1.66
Later Family, Work Break	0.50	−2.12 to 3.20	0.84	−1.87 to 3.14	0.08	−2.46 to 2.69
Work, No Family	1.08	−1.00 to 3.21	0.90	−1.28 to 3.14	0.15	−1.89 to 2.23
Earlier Family, Work Break	−4.22	−6.65 to −1.74	−2.85	−5.41 to −0.22	−2.55	−4.97 to −0.07
Part-time Work, Earlier Family	−4.21	−6.53 to −1.84	−3.19	−5.61 to −0.71	−2.68	−4.97 to −0.32
No Paid Work, Earlier Family	−8.70	−12.93 to −4.26	−5.75	−10.52 to −0.73	−4.93	−9.57 to −0.05
Work, Divorced Parent	−4.05	−7.14 to −0.85	−2.52	−5.79 to 0.87	−2.64	−5.73 to 0.56
Teen parent	−5.60	−11.38 to 0.55	−2.84	−10.62 to 5.61	−2.90	−10.36 to 5.19
Unstable Work, No Family	−5.02	−10.99 to 1.36	−1.68	−9.00 to 6.24	−3.02	−10.05 to 4.56
R^2^ (%)	12.0		13.8		23.8	

**Triglycerides (n=6591)**	**Per cent difference**	**95% CI**	**Per cent difference**	**95% CI**	**Per cent difference**	**95% CI**

Work, Later Family	Ref		Ref		Ref	
Work, Cohabitation, Later Parents	−1.93	−7.92 to 4.45	−4.95	−11.05 to 1.58	−5.29	−11.13 to 0.95
Work, Marriage, Non-Parent	1.08	−4.41 to 6.89	0.47	−5.28 to 6.57	1.23	−4.33 to 7.10
Work, Earlier Family	10.10	5.69 to 14.69	6.39	1.96 to 11.02	3.46	−0.69 to 7.78
Later Family, Work Break	1.70	−4.54 to 8.36	2.54	−3.90 to 9.42	3.92	−2.38 to 10.62
Work, No Family	−1.36	−6.16 to 3.70	−1.42	−6.42 to 3.89	−0.37	−5.26 to 4.76
Earlier Family, Work Break	9.85	3.33 to 16.79	5.61	−0.90 to 12.55	5.04	−1.19 to 11.66
Part-time Work, Earlier Family	7.58	1.47 to 14.06	4.54	−1.63 to 11.05	3.56	−2.30 to 9.77
No Paid Work, Earlier Family	14.31	2.37 to 27.64	4.82	−6.98 to 18.12	2.92	−8.40 to 15.64
Work, Divorced Parent	9.11	0.87 to 18.02	5.10	−3.15 to 14.05	3.99	−3.90 to 12.54
Teen parent	18.76	2.82 to 37.18	7.51	−10.82 to 29.61	5.82	−11.61 to 26.69
Unstable Work, No Family	13.59	−2.92 to 32.90	8.87	−9.85 to 31.49	5.29	−12.65,26.79
R^2^ (%)	14.0		15.2		22.6	

**HbA1c (n=6682)**	**Reg coefficient**	**95% CI**	**Reg coefficient**	**95% CI**	**Reg coefficient**	**95% CI**

Work, Later Family	Ref		Ref		Ref	
Work, Cohabitation, Later Parents	0.11	0.03 to 0.18	0.06	−0.02 to 0.14	0.05	−0.03 to 0.12
Work, Marriage, Non-Parent	0.002	−0.06 to 0.07	0.02	−0.05 to 0.09	0.02	−0.04 to 0.09
Work, Earlier Family	0.12	0.07 to 0.17	0.09	0.04 to 0.14	0.06	0.01 to 0.11
Later Family, Work Break	0.07	−0.003 to 0.15	0.08	0.01 to 0.16	0.09	0.02 to 0.16
Work, No Family	0.03	−0.03 to 0.09	0.04	−0.02 to 0.10	0.05	−0.01 to 0.11
Earlier Family, Work Break	0.05	−0.02 to 0.13	0.04	−0.04 to 0.11	0.02	−0.05 to 0.10
Part-time Work, Earlier Family	0.03	−0.04 to 0.10	0.02	−0.05 to 0.09	0.004	−0.06 to 0.07
No Paid Work, Earlier Family	0.17	0.02 to 0.31	0.14	−0.02 to 0.29	0.06	−0.08 to 0.20
Work, Divorced Parent	0.16	0.06 to 0.25	0.13	0.04 to 0.23	0.12	0.03 to 0.21
Teen parent	0.10	−0.07 to 0.28	0.05	−0.18 to 0.28	0.03	−0.19 to 0.25
Unstable Work, No Family	0.17	−0.01 to 0.36	0.15	−0.07 to 0.37	−0.03	−0.25 to 0.19
R^2^ (%)	1.5		2.0		8.1	

*Adjusted for gender, father's occupational class, child health, educational attainment.

†Adjusted for gender, father's occupational class, child health, educational attainment, household occupational class, smoking status, exercise frequency, problem drinking and BMI.

BMI, body mass index; HbA1c, glycated haemoglobin; HDL, high-density lipoprotein; LCT, life course type; Reg, regression.

#### Educational attainment and childhood predictors

Model 2 in table 2 tests whether the increased metabolic risks associated with earlier parenthood transitions (and, to a lesser extent, weak labour market ties) are due to lower levels of educational attainment and disadvantaged childhood circumstances that set people onto disadvantaged work-family pathways. The attenuating effects of education and early life circumstances were greatest for those in the ‘No Paid Work, Earlier Family’ group. For this group, the inclusion of education and early life factors rendered non-significant all metabolic risks except HDL cholesterol (although the coefficients remained large for triglycerides and HbA1c). The inclusion of education and early life factors also reduced to non-significance the associations between ‘Work, Divorced Parent’ and HDL cholesterol and triglycerides, as well as the elevated triglycerides among the ‘Part-time Work, Earlier Family’, ‘Earlier Family, Work Break’, and ‘Teen parent’ groups (although, again, the coefficients remained large). Education and childhood circumstances did not attenuate the greater metabolic risks found for the ‘Work, Earlier Family’ group, with the exception of HDL cholesterol which became non-significant.

#### Adult mediators

Model 3 in table 2 examines the potential mediating effects of adult health behaviours, household social class and BMI. Raised levels of DBP and triglycerides in the ‘Work, Earlier Family’ group were explained by these mediators (primarily by BMI for both outcomes). Otherwise, higher metabolic risk was not mediated by adult factors. Levels of WC, SBP and HbA1c remained significantly elevated for those in the ‘Work, Earlier Family’ group. WC remained significantly larger for ‘Teen parents’, and HDL cholesterol remained significantly lower for those who combined earlier parenthood with weak labour market trajectories: ‘No Paid Work, Earlier Family’, ‘Part-time Work, Earlier Family’, and ‘Earlier Family, Work Break.’ HbA1c continued to be higher for ‘Work, Divorced Parents’, and the ‘Later Family, Work Break’ group.

Results for HDL cholesterol and triglycerides were unchanged in a sensitivity analysis which removed individuals on lipid-lowering medications. Associations between work-family LCTs and HbA1c were weaker when those taking antidiabetic medication were excluded. This was because HbA1c levels were significantly higher among medication users, and usage was concentrated in the ‘Work, Earlier Family’ and ‘Work, Divorced Parent’ groups.

## Discussion

This study has investigated metabolic risk factors in relation to work-family life courses (to age 42) among men and women in the NCDS British birth cohort. Each of the work-family biographies characterised by earlier transitions to parenthood was associated with significantly elevated metabolic risk factors, regardless of the strength of attachment to paid work or the stability of marriage over the life course. The one exception to this was HDL cholesterol where risk was concentrated in groups characterised by earlier parenthood combined weaker ties to paid work. We examined whether early life factors set individuals onto a ‘chain of risk’ by selecting them into less salutary work-family biographies.^31^ In this cohort, those in work-family groups characterised by earlier parenthood did have lower levels of educational attainment than those in groups who became parents later; however, earlier parents were not significantly more likely than other members of the cohort to have had poor physical or emotional health or live in disadvantaged socioeconomic circumstances in childhood. We investigated whether the increased mid-life health risk among earlier parents in this cohort was explained by the role of education in setting early parents onto a life course of accumulated disadvantage or stress. The inclusion of educational qualifications in the models did attenuate associations between certain groups and certain metabolic risk factor outcomes. Nevertheless, higher metabolic risk persisted for other groups, particularly those who combined earlier parenthood with stable full-time employment and marriage across the life course (to age 42).

We then examined whether the metabolic risk that remained for earlier parenthood groups after accounting for education and early life factors operated through unhealthy behaviours or disadvantaged occupational class in adulthood. Adult mediators (particularly BMI) were important for triglycerides and DBP only. This attenuating role of BMI suggests metabolic risk may be working partly through obesogenic lifestyle factors among earlier parents. A recent British study showed early parenthood to be linked with lower levels of physical activity in later life,^27^ and previous studies have suggested that parents of preschool-aged children are significantly less likely than other parents and non-parents to find time for exercise.^33^ Perhaps if parenthood begins at younger ages, unhealthy behavioural patterns (such as lack of exercise) are established which persist across adulthood.

The metabolic risk among earlier parents that was unexplained suggests mediation through paths not well captured in this study, such as chronic stress related to parenting with fewer financial, emotional and developmental resources, or simply that later parents have had less life course exposure to the stresses of parenthood by midlife. Prior work on the health consequences of age at parenthood has tended to be US-based.^25^ ^28^ However, a previous study of early adult transitions in this British birth cohort showed early parenthood to be associated with higher levels of depressive symptoms at age 33,^29^ and research on an earlier born British birth cohort found an increased risk of coronary heart disease among women and men who became parents at an early age.^26^ Most recently, a longitudinal British study showed early parenthood to be linked with increased allostatic load and long-term illness in later life.^27^ Authors of these studies speculate that younger parents have accumulated less human and social capital to cope with the stresses of parenting. It is important to note that none of the work-family life courses defined in the current study are characterised by older entry into parenthood (beyond age 35) when social and biological processes may work in differing directions.^41^ ^42^ The majority of individuals in our ‘later family’ groups had entered parenthood by age 31, and nearly all (96%) were parents before age 35.

We did not find significant gender differences in associations between work-family LCTs and metabolic risk factors, and this was unexpected. Groups characterised by earlier transitions to parenthood were associated with increased metabolic risk for both men and women. However, there were almost no men in types characterised by weak(er) ties to paid work (including three of the ‘earlier family’ groups), making gender differences in the health risks for these groups difficult to detect.

Previous studies have generally shown worse health profiles for those taking periods of time out of employment to look after the home and family.^13–20^ For example, in an older British cohort, obesity prevalence in mid-life was higher among women who spent long periods out of the labour market to care for children, compared with those who combined stronger ties to paid work with motherhood and a stable marriage.^13^ Similarly, a recent multichannel sequence analysis of work-family LTCs in the USA showed elevated rates of mortality among non-working mothers, particularly those who were single.^20^ In this cohort, not all groups with weak ties to the labour market had elevated metabolic risks; rather, risks tended to be concentrated more specifically among those with weak ties who were *also* earlier parents. The divergence may partly reflect the fact that previous studies have not simultaneously taken account of the timing of parenthood and work breaks.

Limitations of this study include the inability to investigate qualitative aspects of work and family relations (such as satisfaction, trust, reciprocity, support or control). We were also unable to consider resources available for childcare or the extent to which employers accommodate parental responsibilities. Individuals in our LCTs characterised by later transitions to parenthood were less likely be lost to follow-up than individuals from other LCTs. This bias may underestimate the health advantages in this group if less healthy earlier parents were lost to follow-up. Additionally, we did not include comorbidities which might influence metabolic risk factors, although these may represent health outcomes which overlap conceptually with our risk factors, as well as potential precursers of metabolic risk.

On the other hand, this study has a number of strengths. It uses multichannel sequence analysis to simultaneously consider work, partnerships and parenthood histories of both women *and* men throughout the prime working and childrearing years. By including both men and women, we learned that work and family life courses, and particularly the timing of parenthood, are associated with health risk factors for men as well as women. By examining multiple domains simultaneously we were able to show that, for HDL cholesterol in particular, risk was concentrated among those who combined earlier parenthood with weaker ties to paid work. The longitudinal design also allowed for the consideration of prior childhood factors which may confound associations, and demonstrated that metabolic risk persisted after their inclusion, particularly for those who combined earlier parenthood with stable full-time employment. Another strength of this study is that missing data were accounted for using multiple imputation, allowing us to retain as many cases as possible while also ensuring accurate representation of work and family histories. And finally, in contrast to many previous studies linking work and family histories to subjective health, we used objective markers of health. The use of objective biomarkers reduces reporting bias and suggests links between work-family life courses and physical health in addition to any links which may exist with mental health.

In conclusion, this is one of the first studies to link earlier parenthood with objectively measured biological health risk factors in mid-life, independent of health status prior to parenthood. Further work is needed to investigate the causal mechanisms implicated, such as fewer resources to withstand the stresses of parenting among young parents, or greater accumulated exposure to the stresses of parenting. Stresses associated with early parenthood may vary by cohort depending on evolving norms regarding timing of parenthood, changing familial support structures and other sociohistorical context. The cohort in this study entered parenthood later than previous cohorts, but they nevertheless formed their families before the largest shifts towards delayed childbearing and childlessness.^38^ A next step will be to replicate this analysis in the earlier 1946 and recent 1970 British birth cohorts when comparable data become available.

**What is already known on this subject**

- Previous studies have generally found better health among people who combine paid work with family responsibilities; however, this work has generally excluded men and relied on subjective measures of health measures and information about work and family from only one or two time points in the life course.

**What this study adds**

- Metabolic risk factors were investigated in relation to holistic work-family life courses (to age 42) among men and women in the National Child Development Study 1958 British birth cohort. Life courses characterised by earlier transitions into parenthood (when cohort members were in their teens or early twenties) were associated with significantly increased metabolic risk in middle age, regardless of the strength of their attachment to paid work or the stability of their marriage over the life course. These associations were only partially attenuated by educational qualifications, early life circumstances or adult mediators, and associations between work-family life course types and metabolic risk factors did not differ significantly by gender.

## Footnotes

i BMI was not included in models predicting WC.
